# Physiological importance and role of Mg^2+^ in improving bacterial resistance to cesium

**DOI:** 10.3389/fmicb.2023.1201121

**Published:** 2023-06-21

**Authors:** Yoshiki Ishida, Chongkai Zhang, Katsuya Satoh, Masahiro Ito

**Affiliations:** ^1^Graduate School of Life Sciences, Toyo University, Oura-gun, Gunma, Japan; ^2^Faculty of Life Sciences, Toyo University, Oura-gun, Gunma, Japan; ^3^Department of Quantum-Applied Biosciences, Takasaki Institute for Advanced Quantum Science, Foundational Quantum Technology Research Directorate, National Institutes for Quantum and Radiological Science and Technology, Takasaki, Gunma, Japan; ^4^Bio-Resilience Research Project (BRRP), Toyo University, Oura-gun, Gunma, Japan; ^5^Bio Nano Electronics Research Centre, Toyo University, Kawagoe, Saitama, Japan

**Keywords:** cesium-resistant microorganism, magnesium, *Microbacterium*, *Bacillus subtilis*, ribosome

## Abstract

Cesium (Cs) is an alkali metal with radioactive isotopes such as ^137^Cs and ^134^Cs. ^137^Cs, a product of uranium fission, has garnered attention as a radioactive contaminant. Radioactive contamination remediation using microorganisms has been the focus of numerous studies. We investigated the mechanism underlying Cs^+^ resistance in *Microbacterium* sp. TS-1 and other representative microorganisms, including *Bacillus subtilis*. The addition of Mg^2+^ effectively improved the Cs^+^ resistance of these microorganisms. When exposed to high concentrations of Cs^+^, the ribosomes of Cs^+^-sensitive mutants of TS-1 collapsed. Growth inhibition of *B. subtilis* in a high-concentration Cs^+^ environment was because of a drastic decrease in the intracellular potassium ion concentration and not the destabilization of the ribosomal complex. This is the first study demonstrating that the toxic effect of Cs^+^ on bacterial cells differs based on the presence of a Cs^+^ efflux mechanism. These results will aid in utilizing high-concentration Cs^+^-resistant microorganisms for radioactive contamination remediation in the future.

## 1. Introduction

After the Fukushima nuclear power plant accident in 2011, a large amount of radioactive cesium (Cs), ^134^Cs and ^137^Cs, was released into the environment, resulting in serious impacts on human health and the environment (Hirose, 2016; Nakamura et al., 2022; Wu et al., 2022). Since then, studies on contamination of the soil with radioactive Cs ions (Cs^+^), decontamination and bioremediation efforts, and the quest to identify Cs^+^-tolerant microorganisms have been prominent. Imparting radio resistance to Cs^+^-resistant microorganisms would lead to the creation of highly functional radiation-tolerant bacteria, which could efficiently recover radioactive Cs from radioactive Cs-contaminated environments. Therefore, creating highly functional microorganisms to be used for bioremediation in environments contaminated with radioactive Cs is desirable.

Cs, an alkali metal, generally exists as a monovalent cation in solutions. Radioactive isotopes of Cs are water-soluble and can be taken up by the ion uptake system of living organisms. The isotopes ^134^Cs and ^137^Cs have long half-lives (^134^Cs, 2.0652 years; ^137^Cs, 30.1 years), are discharged into the external environment during nuclear power generation and emit γ- and β-rays when they decay (Hampton et al., 2004; Sanial et al., 2017). In addition, as Cs^+^ possess physicochemical properties similar to those of potassium ions (K^+^), Cs^+^ is transported into the cells through the K^+^ transport system (Perkins and Gadd, 1995). Since *Escherichia coli* does not have a Cs^+^ efflux system, Cs^+^ accumulates inside its cell. K^+^ is excreted from the cell by the K^+^ efflux system instead of Cs^+^. As a result, intracellular K^+^ concentration is significantly reduced (Jung et al., 2001). In addition, K^+^ contributes to the stabilization of ribosomes, and its deficiency results in growth inhibition (Avery, 1995; Hampton et al., 2004).

*Microbacterium* sp. TS-1 (TS-1), an alkaliphilic bacterium, was isolated from a jumping spider (*Salticidae*) in our laboratory (Fujinami et al., 2013). It is a gram-positive aerobic bacterium with an optimum pH range of 8.0–9.0. In addition, it is a high-concentration Cs^+^-resistant bacterium that can grow in media containing 1,200 mM CsCl (Koretsune et al., 2022). Two significant mechanisms of Cs^+^ resistance in TS-1 have been reported (Koretsune et al., 2022; Ishida et al., 2023). The first mechanism works through the Cs^+^/H^+^ antiporter CshA, which excretes Cs^+^ from the cell and maintains a low intracellular Cs^+^ concentration. The Cs^+^-sensitive mutant strain Mut4 is a CshA defective mutant isolated previously (Koretsune et al., 2022). The second mechanism involves maintaining Cs^+^ resistance through the uptake of magnesium ions (Mg^2+^) into cells via the magnesium transporter MgtE, even when the Cs^+^ concentration increases (Ishida et al., 2023). A mutation in the *mgtE* gene of TS-1 (mutant strain Mut5) leads to Cs^+^-sensitivity because of the deficiency of intracellular Mg^2+^ (Ishida et al., 2023), suggesting that Mg^2+^ plays an essential role in Cs^+^ resistance in TS-1. Magnesium is a group 2 element and an essential trace element for organisms (Fiorentini et al., 2021). It plays various roles, such as maintaining the structure of ribosomes and enzymatic activity (Akanuma et al., 2014; Pontes et al., 2016). If the Mg^2+^ transporter MgtE contributes toward improving Cs^+^ resistance in TS-1, it would be interesting to investigate whether Mg^2+^ uptake into cells also leads to the enhancement of Cs^+^ tolerance in other microorganisms. These strains have multiple Mg^2+^ transporters but no Cs^+^/H^+^ antiporter CshA homologous proteins.

Therefore, to verify whether Mg^2+^ plays an essential role in Cs^+^ resistance in microorganisms, we used the gram-positive bacteria *Bacillus subtilis* BR151MA (wild-type), *Staphylococcus aureus* IAM12544, gram-negative *E. coli* W3110, and *Pseudomonas aeruginosa* NBRC13275 as representative microorganisms. In addition, the eukaryote *Saccharomyces cerevisiae* JCM1499 was used for Cs^+^-resistant growth testing with MgCl_2_ added to the medium.

Functional analysis of TS-1 showed that in an environment with a Cs^+^ concentration of ≤200 mM, Cs^+^ resistance was maintained by increasing the Mg^2+^ concentration in cells. Conversely, in a high Cs^+^ outer environment of >200 mM, Cs^+^ resistance was maintained with a low Cs^+^ concentration in cells through CshA. However, the exact mechanism by which the increase in cell Mg^2+^ concentration affects Cs^+^ resistance of TS-1 and the underlying physiological functions of CshA maintaining intracellular Cs^+^ concentration remain unknown.

Mg^2+^ stabilizes ribosomal complexes (Hsiao et al., 2009; Nierhaus, 2014; Akanuma et al., 2018). In *B. subtilis*, intracellular Mg^2+^ is stored in ribosomes and contributes to the stabilization of complex ribosomal formation (Akanuma et al., 2014, 2018). The intracellular accumulation of Cs^+^ in *E. coli* reduces intracellular K^+^ concentrations and inhibits growth (Bossemeyer et al., 1989; Avery, 1995). Based on these findings, in this study, we considered the intracellular actions of the Cs^+^ resistance mechanism in TS-1 and hypothesized that in an environment with a Cs^+^ concentration < 200 mM, ribosomes are destabilized when Cs^+^ is taken up into the cells. As Mg^2+^ stabilizes the ribosomal complex, Cs^+^ resistance is acquired by enhancing the Mg^2+^ uptake by MgtE. In a Cs^+^ environment >200 mM, Cs^+^ resistance is achieved by excreting intracellular Cs^+^ through CshA to prevent intracellular accumulation of Cs^+^ and decrease intracellular K^+^ concentration.

Therefore, we analyzed the ribosomal complex to verify the hypothesis regarding the acquisition of Cs^+^ resistance by incorporating Mg^2+^ into TS-1. To investigate the role of Mg^2+^ in Cs^+^ resistance, we attempted to measure changes in Cs^+^, K^+^, and Mg^2+^ concentrations in the cells. Through these experiments, we attempted to elucidate the effects of Cs^+^ concentration on TS-1 and also on *B. subtilis*, in which the relationship between ribosomes and Mg^2+^ has been well studied (Akanuma et al., 2014; Nierhaus, 2014; Akanuma et al., 2018; Lee et al., 2019; Akanuma, 2021).

## 2. Materials and methods

### 2.1. Bacterial strains, growth media, and conditions for culture

The bacterial strains used in this study are listed in Table 1. *B. subtilis* BR151MA (Henkin et al., 1991), *S. aureus* JCM20624, *E. coli* W3110, *P. aeruginosa* IFO13275, and *S. cerevisiae* JCM1499 were used to assess improvements in Cs^+^ resistance. Luria-Bertani (LB), Miller medium (2 mL; BD Difco^™^; BD Biosciences, Franklin Lakes, NJ, United States) (pH 7.5 for *B. subtilis*, pH 7.0 for others) was dispensed into a 14-mL culture tube, inoculated with each single colony, and cultured with shaking at 37°C and 200 rpm for 8 h. This culture solution was used as the pre-culture. A bioshaker BR-43FM (TAITEC Co., Ltd., Koshigaya, Japan) was used for shaking the culture, and the same equipment was used unless otherwise specified.

**Table 1:** Bacterial strains and plasmids used in the present study.

Strain	Genotype	References
*Microbacterium* sp. TS-1	Wild-type	Fujinami et al. (2013)
Mut4	Cs^+^-sensitive mutant from TS-1, MTS1_00475 (CshA: W253*)	Koretsune et al. (2022)
Mut5	Cs^+^-sensitive mutant from TS-1, MTS1_03028 (MgtE: T396I)	Ishida et al. (2023)
Mut4R	Cs^+^-resistant revertant from Mut4, MTS1_00475 (W253* → *253R) (Intragenic suppression)	Koretsune et al. (2022)
Mut5R	Cs^+^-resistant revertant from Mut5, MTS1_03028 (T396I → I396T) (true reversion)	Ishida et al. (2023)
*Escherichia coli* W3110	F^−^ *IN(rrnD-rrnE1) rph-1*	*E. coli* genetic stock center
*Bacillus subtilis* BR151MA	*lys3, trpC2* (wild-type)	Grundy et al. (1994)
*Staphylococcus aureus* IAM12544	Wild-type	Biological Resource Center, NITE (NBRC), Japan
*Pseudomonas aeruginosa* NBRC13275	Wild-type	Biological Resource Center, NITE (NBRC), Japan
*Saccharomyces cerevisiae* JCM1499	Wild-type	RIKEN Bioresource Research Center, Japan

Various concentrations of MgCl_2_ (25–200 mM final concentration) were added to 2 mL of LB medium supplemented with different concentrations of CsCl (100–600 mM final concentration). Ten microliters of the pre-culture solution were inoculated and cultured with shaking at 37°C and 200 rpm, and the OD_600_ was measured after 16 h. Turbidity was measured using a UV-1800 ultraviolet–visible spectrophotometer (Shimadzu Co., Ltd., Kyoto, Japan). For *S. cerevisiae*, 2 mL of yeast malt (YM) broth (BD Difco^™^; BD Biosciences) was dispensed into a 14-mL capacity culture tube, inoculated using a single colony, and cultured with shaking at 25°C and 200 rpm for 24 h. This was used as the pre-culture. Similar to eubacteria, CsCl and MgCl_2_ were added to the YM broth; approximately 10 μL of the pre-culture was inoculated and cultured with shaking at 25°C and 200 rpm for 24 h, and turbidity was measured at OD_600_.

Alkaliphilic *Microbacterium* sp. TS-1 was grown at 30°C in neutral complex (NC medium) and Tris media (Imazawa et al., 2016). Tris medium consisted of 3.63 g L^−1^ Tris base, 1.47 g L^−1^ citric acid monohydrate, 0.5 g L^−1^ yeast extract, 9 g L^−1^ glucose, and 1% (w/v) trace elements (Cohen-Bazire et al., 1957). Deionized (DI) water was used as the solvent. The pH was adjusted to 8 and 9 using 1 M *N*-methyl-*D*-glucamine. The pH was adjusted to 7 using 5 N sulfuric acid (H_2_SO_4_). Tris medium was used for the monovalent cation resistance test to avoid underestimation of cation influx. The NC medium consisted of 15.5 g L^−1^ K_2_HPO_4_, 4.5 g L^−1^ KH_2_PO_4_, 0.05 g L^−1^ MgSO_4_•7H_2_O, 0.34 g L^−1^ citric acid, 5 g L^−1^ peptone, 2 g L^−1^ yeast extract, 5 g L^−1^ glucose, and 11.7 g L^−1^ NaCl. Deionized water was used as a solvent. The final pH was adjusted to the desired value using KOH or H_2_SO_4_ (Fujinami et al., 2011).

### 2.2. Measurement of intracellular Cs^+^, K^+^, and Mg^2+^ concentration

Intracellular Cs^+^, K^+^, and Mg^2+^ concentrations were measured in TS-1 wild-type, Cs^+^-sensitive mutants (Mut4 and Mut5), and *B. subtilis*. As TS-1 Cs^+^-sensitive mutants and *B. subtilis* did not grow under high Cs^+^ concentration conditions, CsCl was added after culturing. Intracellular ion concentrations were measured 1 h after the addition of CsCl.

Strain TS-1 was inoculated from a single colony by dispensing 2 mL of neutral complex medium (pH 8.0) into a 14-mL culture tube and cultured at 30°C and 200 rpm for 18 h. The culture solution (100 μL) was inoculated into a medieval composite agar medium and pre-incubated at 37°C for 18 h. The cultured neutral complex agar medium was inoculated into 200 mL of 30 mM Tris medium (pH 8.0) such that the turbidity at OD_600_ was 0.1 and cultured at 37°C and 150 rpm. When the turbidity at OD_600_ reached 0.4, 20-mL portions were dispensed, various concentrations of CsCl (0, 100, 200, 300, and 400 mM) were added, and the cells were further cultured for 1 h. The culture solution was centrifuged at 9,100 × *g* at 20°C for 3 min to collect the cells. Unless otherwise specified, the TOMY Seiko MX-305 centrifuge (TOMY SEIKO Co., Ltd., Tokyo, Japan) was used. The supernatant was removed, and the cell pellet was suspended in 5 mL of 300 mM sucrose solution. The suspension was centrifuged at 9,000 × *g* for 3 min at 20°C, and the cell pellet was resuspended in 5 mL of 300 mM sucrose solution. The protein concentration was determined via the Lowry method using a 100-μL aliquot of the suspension. One milligram of protein was used in a volume of 3 μL of bacterial cells. The suspension was then centrifuged at 9,000 × *g* for 3 min at 20°C. The cell pellet was suspended in 5 mL of 5% trichloroacetic acid (TCA) and incubated at 100°C for 10 min. After that, the mixture was centrifuged at 4°C and 9,000 × *g* for 5 min, and the supernatant was used for intracellular ion concentration measurement. The Cs^+^ concentration was measured by taking 1 mL of the intracellular ion concentration measurement sample using an atomic absorption photometer (iCE3400; Thermo Fisher Scientific, Waltham, MA, United States). A 1-mL aliquot was taken from the measurement sample, and the K^+^ concentration was measured using an ANA-135 flame photometer (Tokyo Koden Co., Ltd., Tokyo, Japan). Mg^2+^ concentration was measured using a metalloassay magnesium assay LS kit (Metallogenics Co., Ltd., Chiba, Japan) according to the manufacturer’s instructions. First, 4.5 μL of 5 M NaOH was added to 100 μL of the measurement sample to adjust the pH from 2 to 7.

Subsequently, 3 μL of the pH-adjusted measurement sample was added to 250 mL of the coloring solution, vortexed, and allowed to stand at 20°C for 5 min. Absorbance at OD_660_ was then measured. The Mg^2+^ concentration of the samples was calculated from the obtained value using the following formula:


$$\begin{array}{l} \mathrm{Sample}\;{\mathrm{Mg}}^{2+}\mathrm{concentration}\;\left(\mathrm{mM}\right)=\\ \;\;\;\;\;\;\;\;\;\;\;\;\;\;\;\;\;\;\;\frac{\left(\mathrm{Sample absorbance - blank absorbance}\right)\times 2}{\mathrm{Standard solution absorbance}-\mathrm{blank absorbance}} \end{array}$$

### 2.3. Data analysis

**Figure 1:**
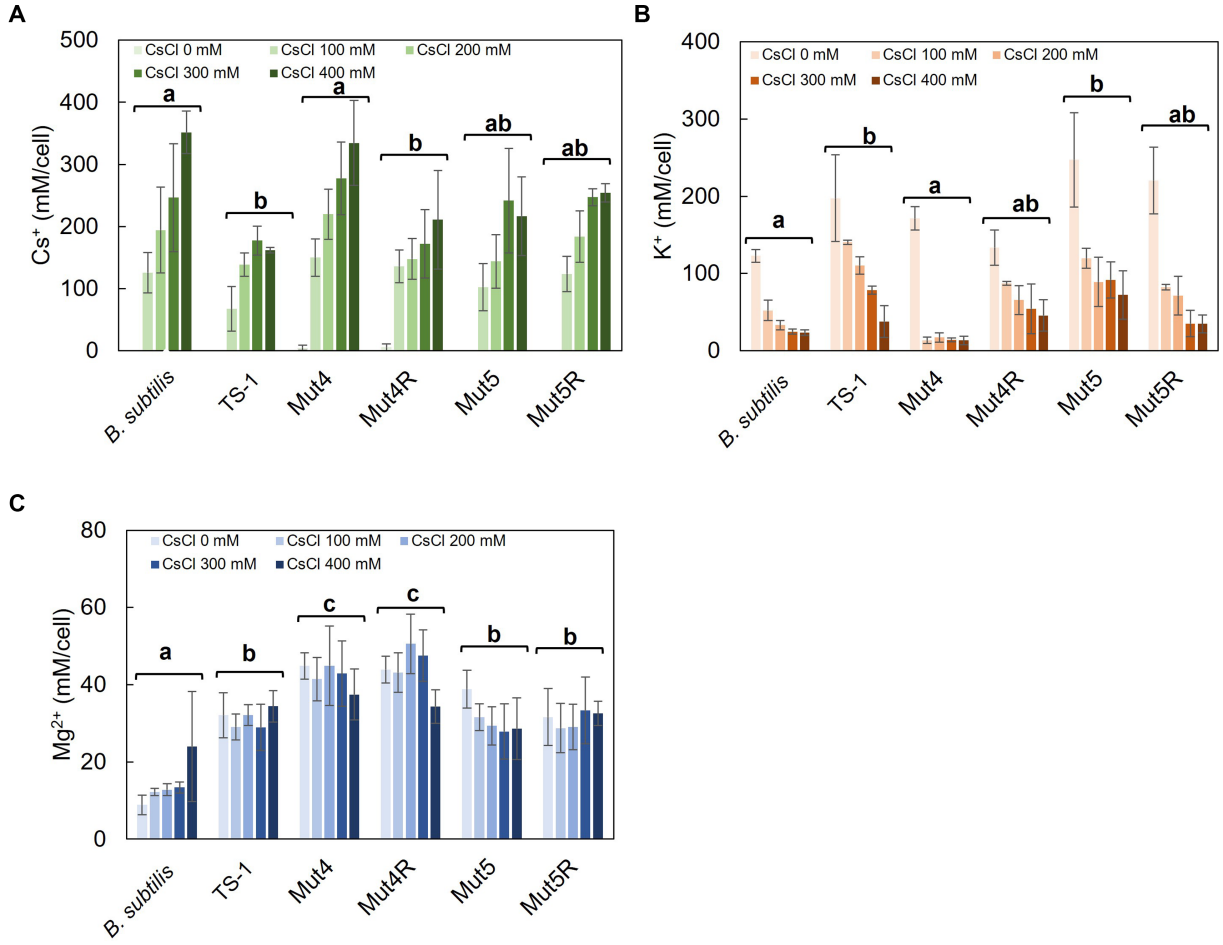
Intracellular Cs^+^, K^+^, and Mg^2+^ concentrations after CsCl treatment in strain TS-1, its mutants, and *B. subtilis.* Strain TS-1 and its derivative mutants were cultured in Tris medium (pH 8.0) and *B. subtilis* in LB medium (pH 7.5) until turbidity reached 0.4. Subsequently, the cells were treated with CsCl for 1 h, collected, and treated with 5% TCA, and the intracellular Cs^+^ concentration **(A)**, intracellular K^+^ concentration **(B)**, and intracellular Mg^2+^ concentration **(C)** were measured. The vertical axis indicates the intracellular concentration (mM/cell), and the series shows the results when each strain was treated with CsCl (0, 100, 200, 300, and 400 mM). Error bars indicate the standard deviation of at least three independent experiments. Different lowercase letters indicate significant differences. In panels **A–C**, multiple comparison analysis was performed between strains after confirming a significant difference by two-way analysis of variance. Different superscript letters (a, b, c) denote significant differences from each other in all combinations.

**Figure 2:**
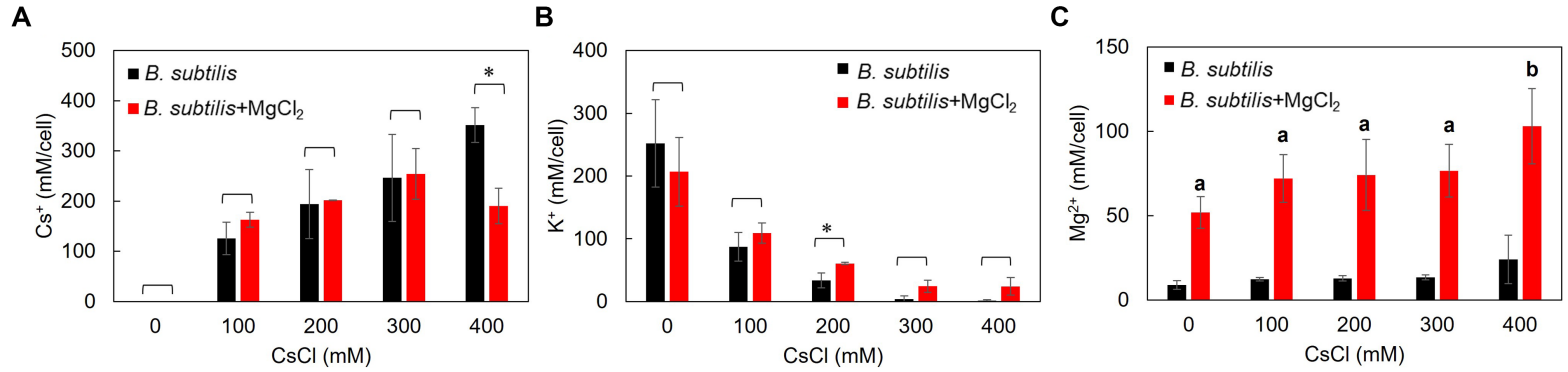
Intracellular Cs^+^, K^+^, and Mg^2+^ concentrations of *B. subtilis* with the addition of CsCl and MgCl_2._ After culturing in LB medium (pH 7.5) to a turbidity of 0.4, the cells were treated with CsCl and MgCl_2_ for 1 h, collected, and suspended in 5% TCA, and the Cs^+^ **(A)**, K^+^ **(B)**, and Mg^2+^ **(C)** concentrations were measured. The vertical axis indicates the intracellular Cs^+^ concentration (mM/cell), and the series shows the results when each strain was treated with CsCl (0, 100, 200, 300, and 400 mM). Error bars indicate the standard deviation of three independent experiments. Asterisk (*) indicates *p* < 0.05, indicating a significant difference. Different superscript letters (a, b) denote significant differences from each other in all combinations.

Statistical analysis was performed with BellCurve for Excel (Social Survey Research Information Co., Ltd., Tokyo, Japan). Tukey test data for *post hoc* analysis of the results in Figures 1, 2 are presented in Supplementary Tables S1, S2, respectively. In Figures 1A–C, multiple comparison analysis was performed between strains after confirming a significant difference by two-way analysis of variance.

### 2.4. Ribosome preparation

Ribosome preparation and fractionation were based on a previously reported method (Takada et al., 2021). Wild-type strain TS-1 and its Cs^+^-sensitive mutants Mut4 and Mut5 were streaked on a pH 8.5 neutral composite agar medium and used as pre-culture (37°C, 18 h). Using an inoculation loop, cells grown on the plate were collected and inoculated (37°C, 200 rpm) in 1,000 mL of Tris medium (pH 8.0). This was used as the culture medium. Cultivation was started at an initial turbidity (OD_600_ = 0.1), and after 4 h of culture initiation, 25 and 50 mL of 8 M CsCl were added. To collect the bacteria, centrifugation (5,000 × *g*, 4°C, 10 min) was performed using an NA-600C rotor (TOMY SEIKO Co. Ltd.) The cells were resuspended in 10 mL Buffer I and subjected to a French press operation twice at 8,000 psi to disrupt the cells. Buffer I (pH 7.6) consisted of 2.42 g L^−1^ tris base, 2.15 g L^−1^ magnesium acetate tetrahydrate, 7.7 g L^−1^ ammonium acetate, 0.015 g L^−1^ ammonium acetate, and 8 mL of 0.1 M phenylmethylsulfonyl fluoride (PMSF). The pH was adjusted to 7.6 with 6 N HCl. Undisrupted cells were removed via centrifugation (12,000 × *g*, 4°C, 15 min), and crude cell debris containing ribosomes in the supernatant was collected. The absorbance of the cell debris at 260 nm was measured using a spectrophotometer and stored at 4°C until ultracentrifugation. Cell lysates were subjected to ultracentrifugation within 3 days of preparation. The same experiment was performed twice to confirm reproducibility.

For *B. subtilis*, 2 mL of LB medium (pH 7.5) was dispensed into a 14-mL culture tube, inoculated using a single colony, and cultured at 37°C and 200 rpm for 8 h. The culture solution (100 μL) was inoculated onto LB agar medium (pH 7.5) and pre-cultured at 37°C for 16 h. The cultured LB agar medium was inoculated into 1 L of LB medium (pH 7.5) such that the turbidity at OD_600_ was 0.03 and cultured at 37°C and 150 rpm. After 2.5 h of culture initiation, CsCl (0, 200, and 400 mM) and 50 mM MgCl_2_ were added, and the mixture was further cultured for 1 h. A crude cell extract was obtained from the culture solution in the same manner as described for strain TS-1.

### 2.5. Ribosome analysis

For strain TS-1 and its Cs^+^-sensitive mutants Mut4 and Mut5, an Ultra-Clear^™^ centrifuge tube (14 mL; Beckman Coulter, Brea, CA, United States) was used for sucrose density gradient ultracentrifugation with 4 mL of 10% sucrose in Buffer I. Thereafter, 4 mL of 35% sucrose in Buffer I was added to the bottom of the tube using a syringe. The upper lid of the tube was covered with parafilm, and the tube was allowed to stand tilted to form a sucrose density gradient at 20°C for 2 h, after which the tube was left at 4°C for 1 h. Crude ribosomal cell extract prepared as described above was layered on top of the sucrose density gradient. An OptimaTML-80XP Ultracentrifuge (Beckman Coulter) was used for ultracentrifugation (222,000 × *g*, 4°C, 3 h) using an SW40Ti rotor (6 × 14 mL; Beckman Coulter). Fractions of 200 μL each were taken from the upper layer of the tube (45 fractions), and the A_260_ of each fraction was measured using a Thermo Nano Drop200C (Thermo Fisher Scientific K.K., Tokyo, Japan). The sucrose concentration was measured using an Atago handheld refractometer (MASTER-PM, ATAGO Co., Ltd., Tokyo, Japan). After the measurements, a separation profile diagram was constructed.

For *B. subtilis*, 4.5 mL of 10% sucrose in Buffer I was poured into a 14-mL Ultra-Clear^™^ centrifuge tube, after which the 4.5 mL of 35% sucrose in Buffer I was added to the bottom of the tube using a syringe. Subsequent experiments were performed as described above for the TS-1 to create a ribosome profile.

## 3. Results

### 3.1. Effect of treatment with or without Mg^2+^ addition on the growth of the representative Cs^+^-resistant microorganisms

**Figure 3:**
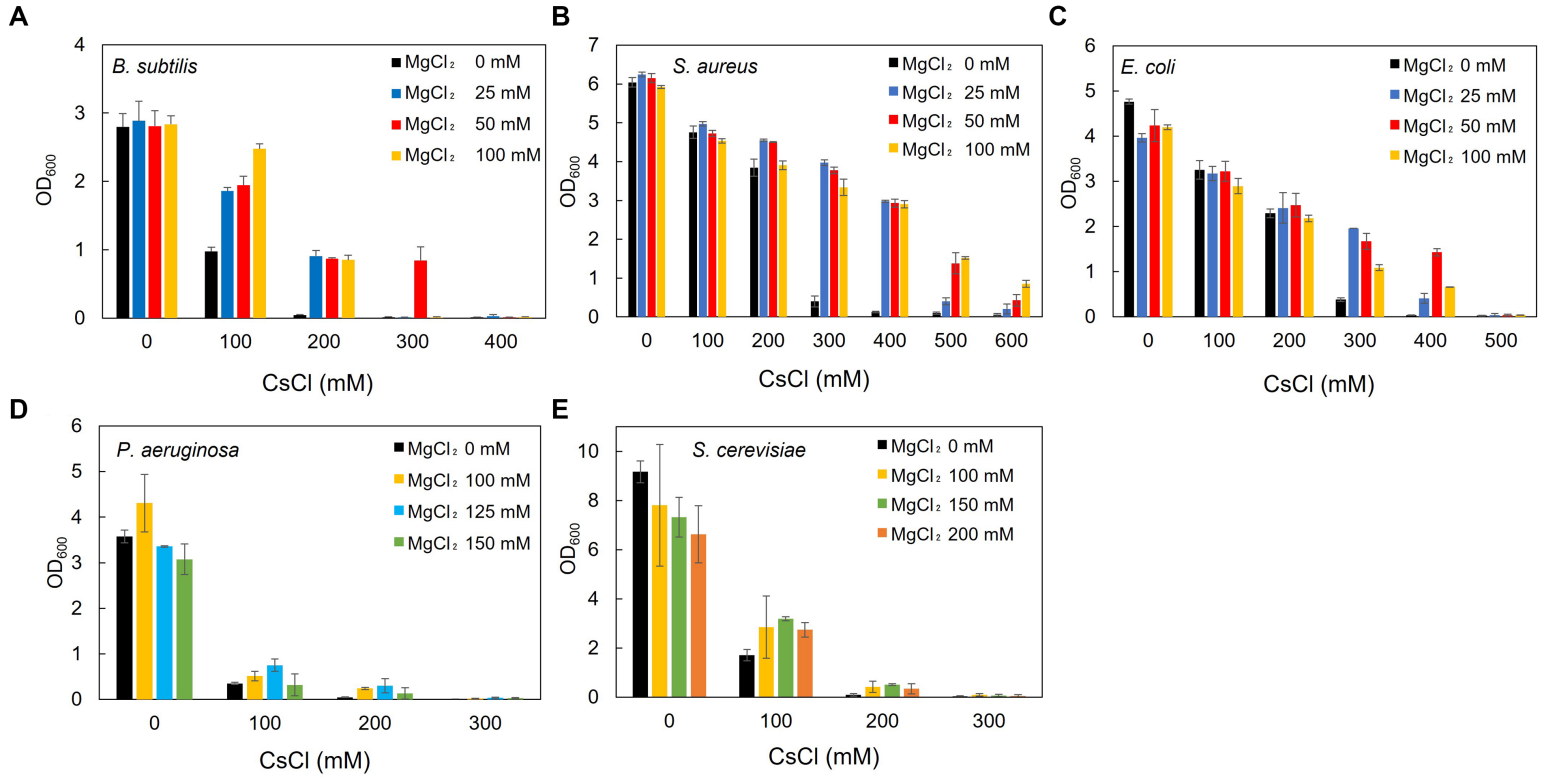
Effect of Mg^2+^ addition on the growth of the representative Cs^+^-resistant microorganisms. *B. subtilis* BR151MA **(A)**, *S. aureus* IAM12544 **(B)**, *E. coli* W3110 **(C)**, and *P. aeruginosa* IFO13275 **(D)** were cultured in LB medium for 16 h, and turbidity at OD_600_ was measured. *B. subtilis* was cultured in an LB medium at pH 7.5 and the others at pH 7.0. *S. cerevisiae* JCM1499 **(E)** was cultured in YM broth for 24 h, and turbidity at OD_600_ was measured. The vertical axis indicates turbidity, and the horizontal axis indicates the CsCl concentration of the medium. Error bars indicate the standard deviation of three independent experiments.

Using *B. subtilis*, *S. aureus*, *E. coli*, *P. aeruginosa*, and *S. cerevisiae* as representative microorganisms, the effect of Mg^2+^ addition on Cs^+^ resistance was investigated. Cs^+^ resistance improved upon the addition of Mg^2+^ to the culture media for all microorganisms, although there was a difference in the degree of Cs^+^ resistance observed between them. *B. subtilis* showed only a slight increase in Cs^+^ tolerance with the addition of Mg^2+^ when the pH of LB medium was 7.0 (data not shown). Therefore, the experiment was performed at pH 7.5, where a more pronounced effect was observed. In *B. subtilis*, Cs^+^ resistance improved to 300 mM (Figure 3A) when 50 mM MgCl_2_ was added to the medium compared with 100 mM without Mg^2+^. Regarding the Cs^+^ resistance of *S. aureus*, growth inhibition was observed at 300 mM CsCl without Mg^2+^. However, growth was observed even with 600 mM CsCl when 100 mM MgCl_2_ was added (Figure 3B). *E. coli* cultures were resistant to 400 mM CsCl when 50 mM MgCl_2_ was added compared with 300 mM without Mg^2+^ (Figure 3C). *P. aeruginosa* grew in the presence of 200 mM CsCl when 125 mM MgCl_2_ was added, compared with only 100 mM CsCl without Mg^2+^ (Figure 3D). *S. cerevisiae* grew in the presence of 200 mM CsCl when 150 mM MgCl_2_ was added, compared to its growth in the absence of Mg^2+^ (Figure 3E).

### 3.2. Comparison of changes in intracellular Cs^+^, K^+^, and Mg^2+^ concentrations in strain TS-1, its Cs^+^-sensitive mutants, its revertants, and *B. subtilis* with and without CsCl treatment

Intracellular Cs^+^ concentrations were measured in TS-1 wild-type, Cs^+^-sensitive mutants (Mut4 and Mut5), revertants (Mut4R and Mut5R), and *B. subtilis* with and without CsCl treatment (Figure 1A). In *B. subtilis*, intracellular Cs^+^ concentration increased with increasing CsCl concentration. Conversely, the intracellular Cs^+^ concentration was maintained at <200 mM in the TS-1 wild-type. In Mut4, the intracellular Cs^+^ concentration increased with increasing CsCl concentration, similar to the trend seen in *B. subtilis*. Mut4R had a lower Cs^+^ concentration than that of Mut4, and this was comparable to the wild-type. Strains Mut5 and Mut5R exhibited intracellular Cs^+^ concentrations intermediate to those of TS-1 wild-type and *B. subtilis*.

Intracellular K^+^ concentrations were measured in TS-1 wild-type, Cs^+^-sensitive mutants, revertants, and *B. subtilis* with and without Cs^+^ treatment (Figure 1B). The addition of CsCl to *B. subtilis* significantly decreased intracellular K^+^ concentration. The TS-1 wild-type showed a more gradual decrease in intracellular K^+^ concentration than that of *B. subtilis* following CsCl addition, showing a significant difference (*p* < 0.05). Similar to *B. subtilis*, Mut4 showed a drastic reduction in the intracellular K^+^ concentration when CsCl was added. Mut4R, Mut5, and Mut5R exhibited approximately similar behaviors as the wild-type strain.

Intracellular Mg^2+^ concentrations were measured in TS-1 wild-type, Cs^+^-sensitive mutants, revertants, and *B. subtilis* with and without Cs^+^ treatment (Figure 1C). There was no significant difference in intracellular Mg^2+^ concentrations between TS-1 wild-type, Mut5, and Mut5R. In contrast, Mut4 and Mut4R showed high intracellular Mg^2+^ concentrations.

Tukey test raw data for *post hoc* analysis of the results in Figure 1 are presented in Supplementary Table S1.

### 3.3. Comparison of Cs^+^ resistance in *B. subtilis* with Mg^2+^ addition and changes in intracellular Cs^+^, K^+^, and Mg^2+^ concentrations

Intracellular Cs^+^ concentrations in *B. subtilis*, representing common microorganisms, were measured with and without CsCl and MgCl_2_ treatments (Figure 2A). At CsCl concentrations of 0–300 mM, adding 50 mM MgCl_2_ did not change the intracellular Cs^+^ concentration, but at 400 mM CsCl, intracellular Cs^+^ concentration decreased by approximately 50%.

Similarly, the intracellular K^+^ concentration in *B. subtilis* was also measured (Figure 2B). There was no significant difference in the intracellular K^+^ concentrations with the addition of 50 mM MgCl_2_ at CsCl concentrations of 0, 100, 300, and 400 mM. However, intracellular K^+^ concentrations significantly increased at a CsCl concentration of 200 mM.

Likewise, intracellular Mg^2+^ concentrations were also measured in *B. subtilis* (Figure 2C). Without CsCl treatment, adding 50 mM MgCl_2_ increased the intracellular Mg^2+^ concentration by approximately 40 mM. As the amount of CsCl increased, the intracellular Mg^2+^ concentration increased slightly, and a significant difference (*p* < 0.05) was observed at a CsCl concentration of 400 mM.

Tukey test raw data for *post hoc* analysis of the results in Figure 2 are presented in Supplementary Table S2.

### 3.4. Effects of CsCl treatment on the growth of TS-1 and *B. subtilis*

**Figure 4:**
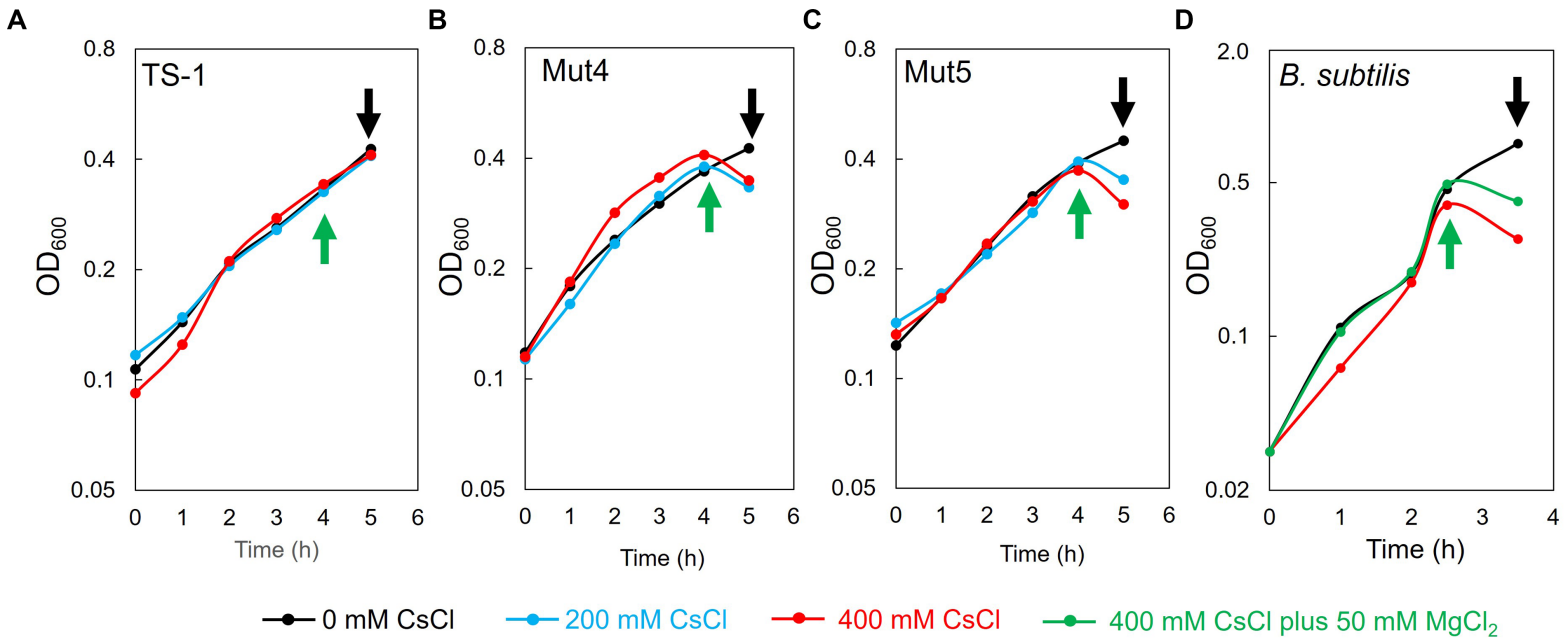
Effects of CsCl treatment on the growth of TS-1 and *B. subtilis.* The horizontal axis indicates culture time (h), and the vertical axis indicates turbidity (OD_600_). For TS-1 **(A)**, Mut4 **(B)**, Mut5 **(C)**, and *B. subtilis* **(D)**, CsCl was added at the time points indicated by the green upward arrow and collected at the time points indicated by the black downward arrow.

TS-1 wild-type, Mut4, and Mut5 were cultured, treated with CsCl after 4 h of culturing, and harvested after 1 h to prepare ribosomes. The doubling times of TS-1 wild-type, Mut4, and Mut5 up to 4 h of culture were approximately 2.6, 2.5, and 2.5 h, respectively. The growth of the TS-1 wild-type continued regardless of the presence or absence of CsCl treatment (Figure 4A). In contrast, growth inhibition of both Mut4 and Mut5 was observed after the addition of CsCl (Figures 4B,C). Next, *B. subtilis* was cultured, CsCl treatment was performed after 2.5 h of culture, and the cells were harvested after 1 h to prepare ribosomes. The doubling time of *B. subtilis* up to 2.5 h before culture was approximately 37 min, and growth inhibition was observed after the addition of CsCl (Figure 4D).

### 3.5. Effects of CsCl treatment in TS-1 on ribosomal complexes

**Figure 5:**
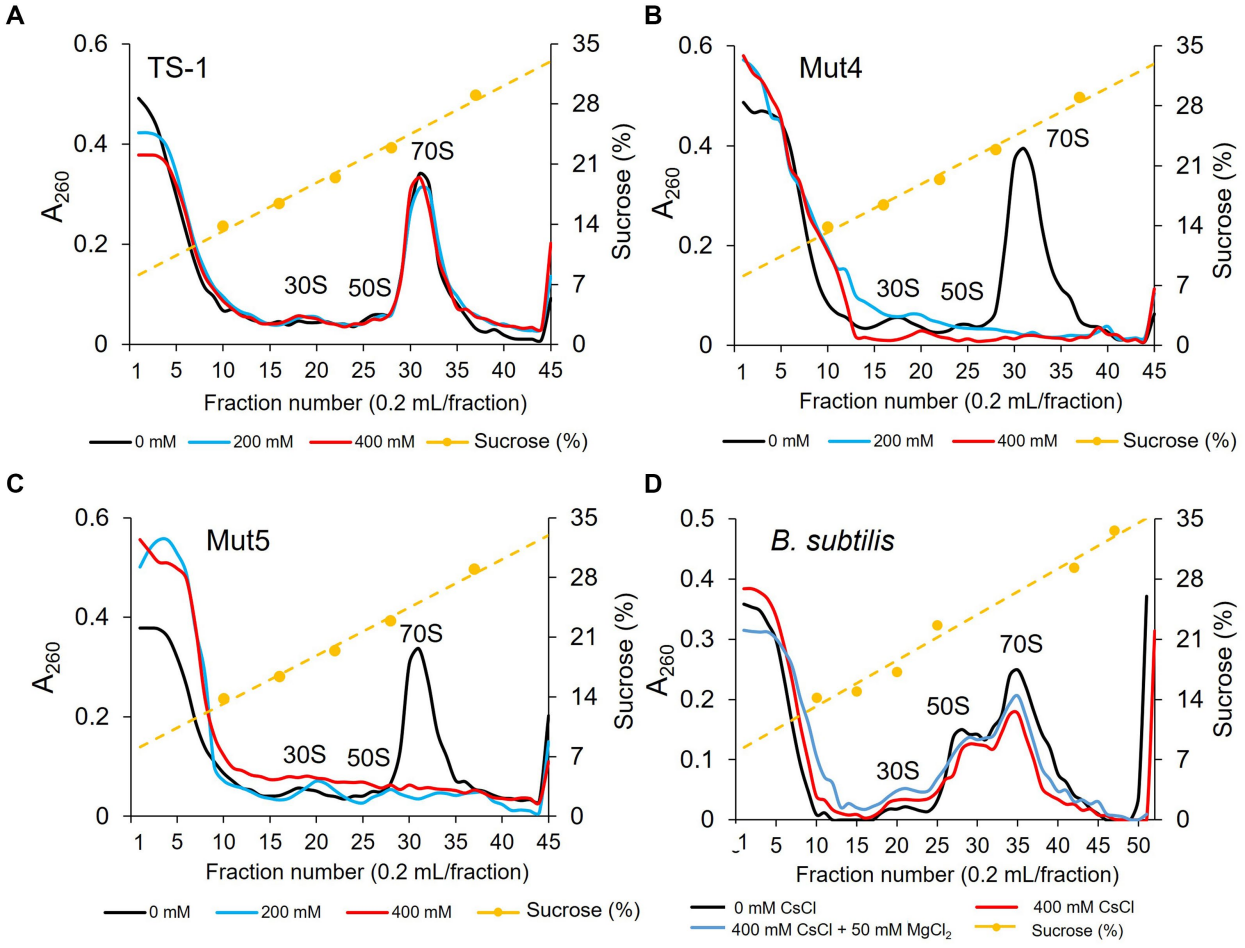
Ribosomal complex profiles with and without CsCl treatment in strain TS-1, its mutants, and *B. subtilis.* The effects of CsCl treatment on complex ribosome formation in strain TS-1 **(A)**, its mutant strains Mut4 **(B)** and Mut5 **(C)**, and *B. subtilis* **(D)** were investigated. Each crude cell extract was obtained and analyzed for ribosomal complexes using sucrose density gradient ultracentrifugation. The first vertical axis indicates the absorbance at A_260_, the second vertical axis shows the sucrose concentration, and the horizontal axis indicates the fraction number. Two independent experiments were performed to confirm reproducibility.

To investigate the effect of Cs^+^ treatment on ribosome complex formation in the TS-1 wild-type and its mutants, crude cell extracts were prepared from cultured cells, and the ribosome complexes were separated using sucrose density gradient ultracentrifugation (Figure 5A). In the TS-1 wild-type, no effect was observed on the formation of ribosomal complexes, regardless of the presence or absence of CsCl treatment. Next, the ribosome complexes were analyzed for the Cs^+^-sensitive mutants, Mut4 and Mut5, in the same manner as the wild-type (Figures 5B,C). Both mutants were treated with CsCl at 200 and 400 mM, upon which the 70S ribosomes collapsed, and a finer peak than that of 30S ribosomes was observed.

### 3.6. Effects of CsCl and MgCl_2_ treatment in *B. subtilis* on ribosomal complexes

The effect of CsCl treatment on ribosome complex formation was investigated in *B. subtilis*. We also investigated whether ribosome complex formation was affected by the addition of MgCl_2_. Ribosome complexes were analyzed using sucrose density gradient ultracentrifugation in the same manner as that described for the TS-1 strain (Figure 5D). Even when *B. subtilis* was treated with CsCl, 70S ribosomes tended to decrease slightly; however, ribosomes were not significantly affected. In addition, no effect on the ribosome complex was observed when MgCl_2_ was added to the culture medium.

## 4. Discussion

### 4.1. Effect of treatment with and without Mg^2+^ addition on the growth of representative microorganisms showing Cs^+^ resistance

Examining the effect of Mg^2+^ addition on Cs^+^ resistance in *B. subtilis*, *P. aeruginosa*, *E. coli*, *S. aureus*, and *S. cerevisiae* as representative microorganisms showed that Cs^+^ resistance was improved by adding Mg^2+^ to the medium for all the five strains tested in this study. Furthermore, it was effective not only for TS-1 but also for microorganisms in general.

Mg^2+^ plays various essential roles in many organisms, including maintaining ribosome structure, genome stabilization, and enzyme activation (Akanuma et al., 2018). It has been reported that increasing the intracellular Mg^2+^ concentration stabilizes ribosomes in *B. subtilis*, in which the structure of the ribosome is destabilized due to the deletion of a part of the ribosomal protein (Akanuma et al., 2018). Therefore, we hypothesized that Mg^2+^ enhanced Cs^+^ tolerance. Cs^+^ uptake by cells affected ribosome complex formation. As Mg^2+^ stabilized the structure of ribosomes, the upper limit of Cs^+^ concentrations tolerated by the strain was considered to be improved.

Differences were found in the improvement in Cs^+^ tolerance and the amount of Mg^2+^ required for the outcome obtained by adding Mg^2+^ to each microbial species culture. It was speculated that the increase in intracellular Mg^2+^ could counteract the drastic decrease in intracellular K^+^ concentration to some extent or that the stability of ribosomes might differ among microorganisms or both.

### 4.2. Comparison of changes in intracellular Cs^+^, K^+^, and Mg^2+^ concentrations in strain TS-1, its Cs^+^-sensitive mutants, its revertants, and *B. subtilis* with and without CsCl treatment

Changes in intracellular Cs^+^, K^+^, and Mg^2+^ concentrations in strains TS-1 and *B. subtilis* with and without CsCl treatment were measured.

In *B. subtilis*, intracellular Cs^+^ concentration increased with increasing CsCl concentration in the medium, and the K^+^ concentration decreased sharply. From the Cs^+^-tolerant growth test results, the intracellular K^+^ concentration decreased to approximately 13% of that in the absence of Cs^+^ at 200 mM CsCl, at which *B. subtilis* could not grow. It was speculated that the extreme decrease in intracellular K^+^ concentration in *B. subtilis* impeded the maintenance of vital functions. This was consistent with results reported for *E. coli* (Avery, 1995). *B. subtilis* and *E. coli* do not harbor a Cs^+^ efflux mechanism. Thus, it was inferred that a decrease in intracellular K^+^ concentration was the leading cause of growth inhibition in these bacteria. CsCl treatment slightly increased intracellular Mg^2+^ but did not make a significant difference. Contrastingly, in strain TS-1, the intracellular Cs^+^ concentration remained lower than that in *B. subtilis* even when the CsCl concentration increased, and the concentration was maintained at approximately <200 mM. Intracellular K^+^ concentration was moderately decreased in TS-1 following Cs^+^ treatment compared to that in *B. subtilis*, and intracellular K^+^ concentration was high compared to that in *B. subtilis*. This may be because TS-1 harbors a Cs^+^ efflux system, CshA (Koretsune et al., 2022). The apparent *K*_m_ value of CshA at pH 8.0 was approximately 250 mM, indicating that TS-1 prevented the intracellular Cs^+^ concentration from increasing and K^+^ deficiency.

Analysis of the changes in intracellular Cs^+^, K^+^, and Mg^2+^ concentrations with and without Cs^+^ treatment in the TS-1 Cs^+^-sensitive mutants and its revertants revealed that CsCl treatment of Mut4 resulted in an influx of Cs^+^ into cells, and the K^+^ concentration was significantly reduced. This was similar to the results obtained for *B. subtilis*, which does not possess a Cs^+^ efflux system. This suggested that Cs^+^ excretion by CshA was essential for Cs^+^ resistance in TS-1 cells. In addition, the intracellular Mg^2+^ concentration in Mut4 was maintained at higher levels than those in the wild-type strain. This suggested that Mg^2+^ was incorporated into cells to resist the toxicity caused by the influx of a large amount of Cs^+^ into the cells. Since CshA in the Cs^+^ efflux system is active in Mut5, which is a MgtE mutant, Cs^+^ and K^+^ concentrations were similar to those of the wild-type even after Cs^+^ treatment. No significant difference was observed in the intracellular Mg^2+^ concentration. This suggested the possibility that another Mg^2+^ transporter performed the uptake of Mg^2+^ into the cell and functioned when Cs^+^ entered the cells of Mut5.

Mg^2+^ has been shown to improve Cs^+^ resistance in several microorganisms. We investigated changes in intracellular Cs^+^, K^+^, and Mg^2+^ concentrations when MgCl_2_ was added simultaneously with CsCl in *B. subtilis*. The addition of 50 mM of MgCl_2_ decreased the intracellular Cs^+^ concentration at a CsCl concentration of 400 mM. The *E. coli* Cs^+^ uptake system, Kup, becomes less active when the cell is filled with K^+^ (Dosch et al., 1991). Although the details of the Cs^+^ uptake system of *B. subtilis* have not been elucidated, the K^+^ uptake system is expected to take up Cs^+^. A significant increase in the intracellular K^+^ concentration was observed when Mg^2+^ was added at a CsCl concentration of 200 mM (*p* = 0.03). Although there was no significant difference in this experiment, the intracellular K^+^ concentration increased at CsCl concentrations of 300 and 400 mM at a level close to the CsCl concentration of 200 mM (300 mM: *p* = 0.06, 400 mM: *p* = 0.08). This was presumed to be because the addition of high concentrations of CsCl and MgCl_2_ enhanced the uptake of K^+^ to adapt to the high osmotic pressure environment.

### 4.3. Effects of CsCl treatment of TS-1, its Cs^+^-sensitive mutants, and *B. subtilis* on ribosomal complexes

To elucidate the mechanism of Cs^+^ resistance through Mg^2+^ uptake in TS-1, we investigated whether the presence or absence of CsCl treatment in TS-1, its Cs^+^-sensitive mutants, and *B. subtilis* affected the formation of ribosomal complexes. In addition, we investigated whether the ribosomal complex was affected when Mg^2+^ was added to *B. subtilis* cultures to improve Cs^+^ tolerance. We observed that Cs^+^ treatment did not affect the ribosomal complex in the TS-1 wild-type. However, in the Cs^+^-sensitive strains Mut4 and Mut5, the 70S ribosomes collapsed following CsCl treatment. However, CsCl treatment did not affect the ribosomal complex of *B. subtilis*.

Mut4 is a CshA mutant, and a large amount of Cs^+^ accumulated in the cells based on the measurement results of intracellular Cs^+^ concentration. Therefore, it is suggested that high concentrations of intracellular Cs^+^ destabilize ribosomes. Strain Mut5 is an MgtE mutant, and although it has no issue with Mg^2+^ uptake, it is presumed that Mg^2+^ uptake cannot be enhanced when Mg^2+^ is required. Therefore, it was speculated that ribosomes destabilized by Cs^+^ could be stabilized owing to the lack of Mg^2+^. High concentrations of K^+^ inhibit ribosome complex formation by competing with Mg^2+^ (Nierhaus, 2014; Senbayram et al., 2015). Because Cs^+^ has physicochemical properties similar to those of K^+^, it was speculated that Cs^+^ destabilized ribosomes by competing with Mg^2+^.

TS-1 wild-type suppressed intracellular Cs^+^ to ≤200 mM in a high-concentration Cs^+^ environment by excreting intracellular Cs^+^ using CshA. In a Cs^+^ environment of ≤200 mM, MgtE is considered to enhance Mg^2+^ uptake and stabilize the ribosomal complex, thereby adapting to the Cs^+^ environment.

*Bacillus subtilis* did not exhibit Cs^+^ resistance, and when treated with CsCl, Cs^+^ accumulated in the cells, similar to Mut4. Thus, the ribosome complex is expected to be similarly destroyed by CsCl treatment. However, CsCl treatment did not have a significant effect on ribosomal complexes. The impact of improving Cs^+^ tolerance by adding Mg^2+^ differed depending on the microbial species. Hence, the stability of ribosomes to Cs^+^ was expected to vary depending on the microbial species. Certain ribosomal proteins function to replace Mg^2+^ for ribosomal stabilization (Hsiao et al., 2009; Akanuma et al., 2014). Although Cs^+^ competes with Mg^2+^ to destabilize ribosomes, it does not affect ribosome stabilization by ribosomal proteins, suggesting that *B. subtilis* ribosomes are not affected by Cs^+^. However, it has been suggested that the influx of Cs^+^ into the cells of *B. subtilis* leads to an extreme decrease in K^+^ and that a shortage of K^+^ interferes with life support.

## 5. Conclusion

In the present study, it was found that Cs^+^ treatment of *B. subtilis* increased the intracellular Cs^+^ concentration, similar to the trend observed in Mut4; therefore, it was presumed that the ribosomal complex would also be disrupted, but no significant effect was observed. This suggests that the degree of cell dependence on Mg^2+^ in the context of the strength of the ribosomal complex differs depending on the microbial species.

In TS-1, when the intracellular Cs^+^ concentration is ≤200 mM, the Cs^+^ that flows into the cell competes with Mg^2+^ and destabilizes the ribosome. Therefore, we inferred that the stability of the ribosomal complex was enhanced by maintaining a high intracellular Mg^2+^ concentration.

Prospects for these results include the application of the Cs^+^/H^+^ antiporter CshA in bioremediation using Mg^2+^ as an additive. Because CshA is a low-affinity antiporter (apparent *K*_m_ value of 250 mM for Cs^+^), it is not suitable for recovering low concentrations of Cs^+^. Therefore, its affinity should be further improved. We plan to elevate the affinity of CshA for Cs^+^ by molecular evolution engineering, such as error-prone PCR. In addition, when using microorganisms that accumulate Cs^+^ but have low Cs^+^ resistance for bioremediation, using Mg^2+^ as an additive is expected to enhance the Cs^+^ resistance of microorganisms and increase the efficiency of their recovery.

## Data availability statement

The original contributions presented in the study are included in the article/Supplementary material, further inquiries can be directed to the corresponding author.

## Author contributions

MI designed the research and wrote the manuscript. YI, CZ, KS, and MI conducted the research. YI, CZ, and MI analyzed the data. All authors contributed to the article and approved the submitted version.

## Funding

This work was supported by a grant for the Toyo University Top Priority Research Promotion Program and the Toyo University intellectual property practical application promotion program.

## Conflict of interest

The authors declare that the research was conducted in the absence of any commercial or financial relationships that could be construed as a potential conflict of interest.

## Publisher’s note

All claims expressed in this article are solely those of the authors and do not necessarily represent those of their affiliated organizations, or those of the publisher, the editors and the reviewers. Any product that may be evaluated in this article, or claim that may be made by its manufacturer, is not guaranteed or endorsed by the publisher.
