# Supplementary material for: Physiological importance and role of Mg2+ in improving bacterial resistance to cesium
Source: Front Microbiol. 2023 Jun 21;14:1201121. doi: 10.3389/fmicb.2023.1201121 (PMC10321302; doi:10.3389/fmicb.2023.1201121)
Supplement: Supplementary file 1 [file Data_Sheet_1.PDF]

Supplementary Material

Physiological Importance and Role of Mg^{2+} in Improving Bacterial Resistance to Cesium

Yoshiki Ishida[1], Chongkai Zhang[2], Katsuya Satoh[3], and Masahiro Ito[1,2,4,5*]

[1]Graduate School of Life Sciences, Toyo University, Oura-gun, Gunma 374-0193 Japan

[2]Faculty of Life Sciences, Toyo University, Oura-gun, Gunma 374-0193 Japan

[3] Department of Quantum-Applied Biosciences, Takasaki Institute of Advanced Quantum Science, Foundational Quantum Technology Research Directorate, National Institutes for Quantum and Radiological Science and Technology, Takasaki, Gunma 370-1292, Japan

[4]Bio-resilience research project (BRRP), Toyo University, Oura-gun, Gunma 374-0193 Japan

[5]Bio nano electronics Research Centre, Toyo University, Kawagoe, Saitama 350-8585 Japan

Supplementary Tables S1 and S2.

Supplementary Table S1. Tukey test data for post hoc analysis of the results in Figure 2.

Fig.2

Dispersion Analysis Table

Fig. 2A

Factor	Type III sum of squares	Degree of freedom	Average square	F value	P value	*：P<0.05 **：P<0.01
Factor 1 (strain)	35797.4184	5	7159.4837	6.3493	0.0011	**
Factor 2 (CsCl)	229169.7238	4	57292.4309	50.8093	P < 0.001	**
Measurement error	22551.9605	20	1127.5980			
whole	287519.1027	29				

Fig. 2B

Factor	Type III sum of squares	Degree of freedom	Average square	F value	P value	*：P<0.05 **：P<0.01
Factor 1 (strain)	25136.5515	5	5027.3103	10.0521	P < 0.001	**
Factor 2 (CsCl)	79844.9385	4	19961.2346	39.9125	P < 0.001	**
Measurement error	10002.4882	20	500.1244			
whole	114983.9783	29				

Fig. 2C

Factor	Type III sum of squares	Degree of freedom	Average square	F value	P value	*：P<0.05 **：P<0.01
Factor 1 (strain)	2821.6058	5	564.3212	27.1032	P < 0.001	**
Factor 2 (CsCl)	21.5476	4	5.3869	0.2587	0.9009	
Measurement error	416.4241	20	20.8212			
whole	3259.5776	29				

Multiple comparison analysis testing

Fig. 2A

Method

Tukey

Strain 1	Strain 2	Average 1	Average 2	Difference	Standard deviation	Statistics	P Value	*：P<0.05 **：P<0.01
B. subtilis	TS-1	183.5628	108.9189	74.6439	21.2377	3.5147	0.0229	*
B. subtilis	Mut4	183.5628	202.5804	19.0176	21.2377	0.8955	0.9432	
B. subtilis	Mut4R	183.5628	112.7583	70.8044	21.2377	3.3339	0.0337	*
B. subtilis	Mut5	183.5628	140.9005	42.6623	21.2377	2.0088	0.3719	
B. subtilis	Mut5R	183.5628	161.2985	22.2643	21.2377	1.0483	0.8956	
TS-1	Mut4	108.9189	202.5804	93.6615	21.2377	4.4102	0.0032	**
TS-1	Mut4R	108.9189	112.7583	3.8395	21.2377	0.1808	1.0000	
TS-1	Mut5	108.9189	140.9005	31.9816	21.2377	1.5059	0.6646	
TS-1	Mut5R	108.9189	161.2985	52.3797	21.2377	2.4664	0.1813	
Mut4	Mut4R	202.5804	112.7583	89.8220	21.2377	4.2294	0.0048	**
Mut4	Mut5	202.5804	140.9005	61.6799	21.2377	2.9043	0.0807	
Mut4	Mut5R	202.5804	161.2985	41.2819	21.2377	1.9438	0.4063	
Mut4R	Mut5	112.7583	140.9005	28.1421	21.2377	1.3251	0.7682	
Mut4R	Mut5R	112.7583	161.2985	48.5402	21.2377	2.2856	0.2453	
Mut5	Mut5R	140.9005	161.2985	20.3980	21.2377	0.9605	0.9251	

	Ave.	Mut4	*B. subtilis*	Mut5R	Mut5	Mut4R	TS-1	Symbol
Mut4	202.5804	a	—	—	—	—	—	a
B. subtilis	183.5628	a	a	—	—	—	—	a
Mut5R	161.2985	a	a	b	—	—	—	ab
Mut5	140.9005	a	a	b	b	—	—	ab
Mut4R	112.7583	**	*	b	b	b	—	b
TS-1	108.9189	**	*	b	b	b	b	b
Number of asterisks		2	2	0	0	0	0	

The symbols with a and b are assigned from the one with the largest number of asterisks.

Fig. 2B

Method

Tukey

Strain 1	Strain 2	Average 1	Average 2	Difference	Standard deviation	Statisics	P Value	*：P<0.05 **：P<0.01
B. subtilis	TS-1	51.2470	112.8965	61.6494	14.1439	4.3587	0.0036	**
B. subtilis	Mut4	51.2470	45.8728	5.3742	14.1439	0.3800	0.9988	
B. subtilis	Mut4R	51.2470	77.2534	26.0063	14.1439	1.8387	0.4652	
B. subtilis	Mut5	51.2470	123.9977	72.7506	14.1439	5.1436	P < 0.001	**
B. subtilis	Mut5R	51.2470	88.8068	37.5597	14.1439	2.6555	0.1294	
TS-1	Mut4	112.8965	45.8728	67.0236	14.1439	4.7387	0.0015	**
TS-1	Mut4R	112.8965	77.2534	35.6431	14.1439	2.5200	0.1651	
TS-1	Mut5	112.8965	123.9977	11.1012	14.1439	0.7849	0.9670	
TS-1	Mut5R	112.8965	88.8068	24.0897	14.1439	1.7032	0.5453	
Mut4	Mut4R	45.8728	77.2534	31.3805	14.1439	2.2187	0.2727	
Mut4	Mut5	45.8728	123.9977	78.1248	14.1439	5.5236	P < 0.001	**
Mut4	Mut5R	45.8728	88.8068	42.9339	14.1439	3.0355	0.0622	
Mut4R	Mut5	77.2534	123.9977	46.7443	14.1439	3.3049	0.0358	*
Mut4R	Mut5R	77.2534	88.8068	11.5534	14.1439	0.8168	0.9609	
Mut5	Mut5R	123.9977	88.8068	35.1909	14.1439	2.4881	0.1746	

	Ave.	Mut5	TS-1	Mut5R	Mut4R	*B. subtilis*	Mut4	Symbol
Mut5	123.9977	a	—	—	—	—	—	a
TS-1	112.8965	a	b	—	—	—	—	ab
Mut5R	88.8068	a	b	c	—	—	—	abc
Mut4R	77.2534	*	b	c	c	—	—	bc
B. subtilis	51.2470	**	**	c	c	c	—	c
Mut4	45.8728	**	**	c	c	c	c	c
Number of asterisks		3	2	0	0	0	0	

The symbols with a, b and c are assigned from the one with the largest number of asterisks.

Fig. 2C

Method

Tukey

Strain 1	Strain 2	Average 1	Average 2	Difference	Standard deviation	Statistics	P Value	*：P<0.05 **：P<0.01
B. subtilis	TS-1	14.2574	31.3245	17.0671	2.8859	5.9139	P < 0.001	**
B. subtilis	Mut4	14.2574	42.3165	28.0591	2.8859	9.7228	P < 0.001	**
B. subtilis	Mut4R	14.2574	43.9160	29.6585	2.8859	10.2770	P < 0.001	**

B. subtilis	Mut5	14.2574	31.2665	17.0090	2.8859	5.8938	P < 0.001	**
B. subtilis	Mut5R	14.2574	31.0646	16.8072	2.8859	5.8239	P < 0.001	**
TS-1	Mut4	31.3245	42.3165	10.9920	2.8859	3.8089	0.0121	*
TS-1	Mut4R	31.3245	43.9160	12.5915	2.8859	4.3631	0.0035	**
TS-1	Mut5	31.3245	31.2665	0.0580	2.8859	0.0201	1.0000	
TS-1	Mut5R	31.3245	31.0646	0.2599	2.8859	0.0901	1.0000	
Mut4	Mut4R	42.3165	43.9160	1.5995	2.8859	0.5542	0.9929	
Mut4	Mut5	42.3165	31.2665	11.0500	2.8859	3.8290	0.0116	*
Mut4	Mut5R	42.3165	31.0646	11.2519	2.8859	3.8989	0.0099	**
Mut4R	Mut5	43.9160	31.2665	12.6495	2.8859	4.3832	0.0034	**
Mut4R	Mut5R	43.9160	31.0646	12.8514	2.8859	4.4531	0.0029	**
Mut5	Mut5R	31.2665	31.0646	0.2019	2.8859	0.0699	1.0000	

	Ave.	Mut4R	Mut4	TS-1	Mut5	Mut5R	*B. subtilis*	Symbol
Mut4R	43.9160	a	—	—	—	—	—	a
Mut4	42.3165	a	a	—	—	—	—	a
TS-1	31.3245	**	*	b	—	—	—	b
Mut5	31.2665	**	*	b	b	—	—	b
Mut5R	31.0646	**	**	b	b	b	—	b
B. subtilis	14.2574	**	**	**	**	**	c	c
Number of asterisks		4	4	1	1	1	0	

The symbols with a, b and c are assigned from the one with the largest number of asterisks.

Supplementary Table 2. Post hoc analysis of the results in Figure 3.

Fig. 3A
Method
Ttest

Condition1	Condition 2	Cs conc.	Average 1	Average 2	Standard deviation 1	Standard deviation 2	Statistics	P Value	*：P<0.05 **：P<0.01
B. subtilis	*B. subtilis* +$MgCl_2$	CsCl 0 mM	0.055	0.042	0.028	0.031	0.3033	0.7768	
		CsCl 100 mM	112.691	169.738	32.549	13.298	1.6225	0.2462	
		CsCl 200 mM	157.734	201.959	56.564	0.000	0.7818	0.5162	
		CsCl 300 mM	207.372	257.822	82.108	61.594	0.4915	0.6717	
		CsCl 400 mM	351.569	206.527	24.338	32.872	3.6403	0.0357	*

Fig. 3B
Method
Ttest

Condition1	Condition 2	Cs conc.	Average 1	Average 2	Standard deviation 1	Standard deviation 2	Statistics	P Value	*：P<0.05 **：P<0.01
B. subtilis	*B. subtilis* +$MgCl_2$	CsCl 0 mM	202.764	168.795	4.411	13.686	2.3624	0.1420	
		CsCl 100 mM	71.551	105.646	8.580	19.036	1.6329	0.2441	
		CsCl 200 mM	26.447	61.955	7.710	0.001	4.6054	0.0441	*
		CsCl 300 mM	0.400	25.169	0.400	11.921	2.0766	0.1735	
		CsCl 400 mM	0.000	32.570	0.000	9.248	3.5218	0.0720	

Fig. 3C
Method
Tukey

Condition	Cs conc.	Cs conc.	Average 1	Average 2	Difference	Standard deviation	Statistics	P Value	*：P<0.05 **：P<0.01
B. subtilis	CsCl 0 mM	CsCl 100 mM	8.8637	12.2305	3.3668	6.5696	0.5125	0.9841	
	CsCl 0 mM	CsCl 200 mM	8.8637	12.8047	3.9409	6.5696	0.5999	0.9719	
	CsCl 0 mM	CsCl 300 mM	8.8637	13.3774	4.5137	6.5696	0.6871	0.9548	
	CsCl 0 mM	CsCl 400 mM	8.8637	24.0108	15.1471	6.5696	2.3057	0.2198	
	CsCl 100 mM	CsCl 200 mM	12.2305	12.8047	0.5742	6.5696	0.0874	1.0000	
	CsCl 100 mM	CsCl 300 mM	12.2305	13.3774	1.1469	6.5696	0.1746	0.9998	
	CsCl 100 mM	CsCl 400 mM	12.2305	24.0108	11.7803	6.5696	1.7932	0.4273	
	CsCl 200 mM	CsCl 300 mM	12.8047	13.3774	0.5727	6.5696	0.0872	1.0000	
	CsCl 200 mM	CsCl 400 mM	12.8047	24.0108	11.2061	6.5696	1.7058	0.4720	
	CsCl 300 mM	CsCl 400 mM	13.3774	24.0108	10.6334	6.5696	1.6186	0.5187	
B. subtilis+ $MgCl_2$	CsCl 0 mM	CsCl 100 mM	51.8742	71.9811	20.1068	15.6333	1.2862	0.7050	
	CsCl 0 mM	CsCl 200 mM	51.8742	74.1212	22.2470	15.6333	1.4230	0.6281	
	CsCl 0 mM	CsCl 300 mM	51.8742	76.5688	24.6946	15.6333	1.5796	0.5401	
	CsCl 0 mM	CsCl 400 mM	51.8742	108.5125	56.6383	15.6333	3.6229	0.0299	*
	CsCl 100 mM	CsCl 200 mM	71.9811	74.1212	2.1401	15.6333	0.1369	0.9999	
	CsCl 100 mM	CsCl 300 mM	71.9811	76.5688	4.5878	15.6333	0.2935	0.9981	
	CsCl 100 mM	CsCl 400 mM	71.9811	108.5125	36.5315	15.6333	2.3368	0.2104	
	CsCl 200 mM	CsCl 300 mM	74.1212	76.5688	2.4476	15.6333	0.1566	0.9998	
	CsCl 200 mM	CsCl 400 mM	74.1212	108.5125	34.3913	15.6333	2.1999	0.2545	
	CsCl 300 mM	CsCl 400 mM	76.5688	108.5125	31.9437	15.6333	2.0433	0.3136	

		Ave.	CsCl 400 mM	CsCl 300 mM	CsCl 200 mM	CsCl 100 mM	CsCl 0 mM	Symbol
B. subtilis +$MgCl_2$	CsCl 400 mM	108.5125	a	—	—	—	—	a
	CsCl 300 mM	76.5688	a	b	—	—	—	ab
	CsCl 200 mM	74.1212	a	b	b	—	—	ab
	CsCl 100 mM	71.9811	a	b	b	b	—	ab
	CsCl 0 mM	51.8742	*	b	b	b	b	b
	Number of asterisks		1	0	0	0	0	

The symbols with a and b are assigned from the one with the largest number of asterisks.
